# Targets and management of hypertension in heart failure: focusing on the stages of heart failure

**DOI:** 10.1111/jch.14553

**Published:** 2022-10-04

**Authors:** Huanhuan Miao, Changhong Zou, Shijie Yang, Yook‐Chin Chia, Minh Van Huynh, Guru Prasad Sogunuru, Jam Chin Tay, Tzung‐Dau Wang, Kazuomi Kario, Yuqing Zhang

**Affiliations:** ^1^ Department of Cardiology Fuwai Hospital Chinese Academy of Medical Sciences & Peking Union Medical College Beijing China; ^2^ Department of Medical Sciences School of Medical and Life Sciences Sunway University Bandar Sunway Malaysia; ^3^ Department of Primary Care Medicine Faculty of Medicine University of Malaya Kuala Lumpur Malaysia; ^4^ Department of Internal Medicine University of Medicine and Pharmacy Hue University Vietnam; ^5^ Advanced Heart Failure & Device Therapies, MEDWAY HEART INSTITUTE Chennai Tamil Nadu India; ^6^ College of Medical Sciences Kathmandu University Bharatpur Nepal; ^7^ Department of General Medicine Tan Tock Seng Hospital Singapore Singapore; ^8^ Cardiovascular Center and Divisions of Cardiology and Hospital Medicine Department of Internal Medicine National Taiwan University Hospital Taipei City Taiwan; ^9^ Division of Cardiovascular Medicine Department of Medicine Jichi Medical University School of Medicine Tochigi Japan

**Keywords:** Clinical Management of High Blood Pressure (HBP), heart failure, hypertension

## Abstract

Hypertension is highly prevalent worldwide and is the major risk factor for heart failure (HF). More than half of the patients with HF in Asia suffer from hypertension. According to the 2022 American Heart Association/American College of Cardiology/Heart Failure Society of America HF guideline, there are four stages of HF, including at risk for HF (stage A), pre‐HF (stage B), symptomatic HF (stage C), and advanced HF (stage D). Given the high prevalence of hypertension as well as HF and the stronger association between hypertension and cardiovascular diseases in Asians compared to the west, measures to prevent and alleviate the progression to clinical HF, especially controlling the blood pressure (BP), are of priority for Asian populations. After reviewing evidence‐based studies, we propose a BP target of less than 130/80 mmHg for patients at stages A, B, and C. However, relatively higher BP may represent an opportunity to maximize guideline‐directed medical therapy (GDMT), which could potentially result in a better prognosis for patients at stage D. Traditional antihypertensive drugs are the cornerstones for the management of hypertension at stages A and B. Notably, calcium channel blockers (CCBs) are inferior to other drug classes for the preventing of HF, whereas diuretics are superior to others. For patients at stage C, GDMT is essential which also helps the control of BP. In particular, sodium‐glucose cotransporter‐2 (SGLT2) inhibitors are newer therapies recommended for the treatment of HF and presumably even in hypertension to prevent HF. Regarding patients at stage D, GDMT is also recommended if tolerable and measures should be taken to improve hemodynamics.

## 1 INTRODUCTION

Hypertension is highly prevalent worldwide and is the major risk factor for many cardiovascular diseases (CVDs), including stroke, coronary artery disease and heart failure (HF).^1^ In Asian countries and regions, nonischemic HF and stroke (especially hemorrhagic stroke) are more common than in western countries. Furthermore, the association between hypertension and CVDs seems to be stronger in Asia than in the west.^2^ In particular, hypertension contributes more to the progression of HF than stroke. In an observational study based on a nationwide database in Japan, the population attributable fractions (PAFs) for HF related to stage 1 and stage 2 hypertension were 23.2% and 51.2%, respectively.^3^ Whereas another study in a large Japanese cohort showed that PAFs of hypertension to stroke were 47.5% in men and 31.4% in women.^4^ The prevalence of hypertension in HF patients ranges from 52% to 71% in Asia, and it is the most common comorbidity in HF.^5^

HF continues to be a public health burden with a prevalence of over 23 million around the world, associated with high mortality and high health care costs.^6^ In the early 2000s, a classification approach of HF according to stages of HF was first proposed by the American College of Cardiology/American Heart Association (ACC/AHA) guideline for the evaluation and management of HF^7^, emphasizing the development and progression of the disease. This method of classification was gradually recognized by many regions, and it was updated in a consensus statement of the universal definition and classification of HF and the 2022 AHA/ACC/Heart Failure Society of America (HFSA) guideline for the management of HF recently.^8^, ^9^ It should be noted that once HF has manifested itself with clinical symptoms, the subsequent mortality is increased by 5‐ to 10‐fold compared to similar patients without clinical HF, and once this happens, there is little chance to reverse the process.^10^ Despite excellent advances in the treatment of HF recently, the prognosis of HF remains unsatisfactory. This highlights the importance of the treatment of hypertension and the prevention of HF at an early stage. Interestingly, Asian populations might benefit more from the treatment of hypertension, since the effect of blood pressure (BP) lowering on the prevention of HF and stroke is much greater than on the prevention of coronary artery disease when compared to the west.^2^, ^11^

In this article, focusing on the stages of HF, we aim to review evidence‐based studies and propose the BP targets as well as the management of hypertension for each stage of HF in Asia.

### 1.1 Pathophysiology and diagnosis of hypertensive HF

In terms of the pathophysiologic mechanisms, numerous neurohormonal factors lead to the development of HF from hypertension, including the activation of the sympathetic nervous system and elevated levels of renin and aldosterone. Hypertension causes left ventricular hypertrophy (LVH) and fibrotic changes, ultimately leading to diastolic dysfunction.^12^ When the pressure and volume overload is sustained, cardiac systolic dysfunction might ensue.^12^ The evolution of hypertensive HF can be categorized into 4 degrees:1) hypertension without LVH; 2) asymptomatic hypertension with LVH; 3) symptomatic HF with preserved ejection fraction (HFpEF); 4) symptomatic HF with reduced ejection fraction (HFrEF).^12^ In Asia, hypertension is the most common etiology of HF after ischemic heart disease, accounting for 4%–54.8% of HF patients.^13^ Actually, HFpEF is much more hypertension‐related, whereas HFrEF is more likely a result of coronary artery disease.^14^

The diagnosis of hypertensive HF is based on the classic symptoms and signs of HF in conjunction with long‐standing hypertension and evidence of LVH.^15^ Furthermore, effective control of BP might be able to reverse HF to some extent, which is also helpful for the diagnosis of hypertensive HF.^13^ In clinical practice, the ECG is recommended as the initial evaluation of LVH due to its low cost and convenience. The combination of several ECG LVH criteria (including Cornell voltage, Cornell product, etc.) could significantly improve the sensitivity for the diagnosis of LVH.^16^ Furthermore, left atrial enlargement can be regarded as another clue of hypertensive HF, which might also be detected by P‐wave terminal force in lead V_1_ in ECG.^16^ Echocardiography is the most common method for the diagnosis of LVH. A left ventricular mass index body of more than 115 g/m2 in men and 95 g/m2 in women will indicate LVH.^17^ Both concentric and eccentric LVH patterns are common in these patients. Women or patients with high renin activity are more likely to exhibit concentric LVH, while the opposite is more likely to exhibit eccentric LVH.^13^ Particularly, it should be noted that the development of HF is always multifactorial and it is often difficult to attribute HF solely to hypertension in clinical practice.^18^

### 1.2 Current classifications of HF

There are several classification methods of HF that are based on distinct perspectives, from New York Heart Association (NYHA) functional class to ejection fraction (EF) categories and stages of HF.^8^, ^9^ Classification of HF by left ventricular ejection fraction (LVEF) is crucial because individuals with different LVEF have distinct responses to treatments and numerous clinical trials used it as inclusion criteria.^19^, ^20^ HFrEF and HFpEF were widely accepted by all guidelines in the classification of HF. But, the third subcategory for patients with EF between 40% and 49% has emerged during the past decade, that is, HF with mid‐range EF (HFmrEF) now termed HF with mildly‐reduced EF (HFmrEF).^9^, ^21^, ^22^ Interestingly, a new subcategory named HF with improved EF (HFimpEF) was proposed in the consensus statement of the universal definition and classification of HF, defining HF with a baseline EF ≤ 40%, a ≥ 10% increase from baseline EF, and a second measurement of EF > 40%.^8^ This is because the changes in EF are associated with the prognosis as well as subsequent treatment. Nonetheless, the classification approach based on EF has its limitations. The accuracy of measurement of EF is questionable as there is great variability among operators. Furthermore, EF can vary over time in different hemodynamic conditions.

The ACC/AHA stages of HF were initially introduced in 2001, including four stages (Table 1): stage A, stage B, stage C, and stage D.^7^ The classification approach was revised and updated in the 2022 AHA/ACC/HFSA guideline for the management of HF (Table 1): at risk for HF (stage A), pre‐HF (stage B), symptomatic HF (stage C), and advanced HF (stage D).^9^ With the introduction of new terminologies, the characteristics of each stage were fully presented, such as “at risk for HF” for stage A and “pre‐HF” for stage B. The new classification approach significantly enlarged the scope of stage A and stage B. Apart from traditional risk factors, patients with genetic variants for cardiomyopathy will be categorized as stage A. Furthermore, individuals with evidence of increased filling pressure and abnormal cardiac biomarkers but without symptoms will be included in stage B. These changes attach great importance to the stages before the onset of symptomatic HF. The primary prevention of HF is vital for public health, given the largest number of patients being at stage A and B.^23^ In summary, the ACC/AHA stages of HF emphasize the development and progression of HF, which plays an important role in guiding the prevention and management of clinical HF.

**TABLE 1:** Stages of heart failure

Stage	Description according to the 2001 ACC/AHA guidelines for HF^7^	Description according to the 2022 AHA/ACC/HFSA guideline for the management of HF^9^
Stage A	Patients at high risk for HF but without structural heart disease or symptoms of HF.	**At risk for HF**:Patients at risk for HF but without symptoms, structural heart disease, or cardiac biomarkers of injury or stretch.
Stage B	Patients with structural heart disease but without symptoms.	**Pre‐HF**:Patients with no symptoms or signs of HF but with evidence of 1 of the following: structural heart disease, evidence of increased filling pressures or patients with risk factors and increased levels of B‐type natriuretic peptides or persistently elevated cardiac troponin in the absence of several conditions.
Stage C	Patients with structural heart disease and prior or current symptoms.	**Symptomatic HF**: Patients with structural heart disease with prior or current symptoms of HF.
Stage D	Patients with refractory HF requiring specialized intervention.	**Advanced HF**: Patients with marked symptoms that interfere with daily life and lead to recurrent hospitalizations despite attempts to optimize guideline‐directed medical therapy.

Abbreviations: ACC/AHA, American College of Cardiology/American Heart Association; AHA/ACC/HFSA, American Heart Association/American College of Cardiology/Heart Failure Society of America; HF, heart failure.

### 1.3 BP targets according to stages of HF

Given the great association between hypertension and HF in Asia, it is imperative to take measures to achieve optimal BP control, which may prevent the development of HF, palliate the progression of the disease and further improve the prognosis of HF. Based on evidence from meta‐analysis, clinical trials as well as observational studies, we aim to propose specific BP targets for each stage of HF in Asia.

For patients at stage A, those with hypertension but without structural heart disease, intensive BP treatment may be beneficial. The Systolic Blood Pressure Intervention Trial (SPRINT) showed that compared with a systolic blood pressure (SBP) target of less than 140 mmHg (1 mmHg = .133kPa), targeting a SBP of less than 120 mmHg significantly reduced the rate of HF by 38%.^24^ The conclusion could even be extended to patients aged ≥ 75 years.^25^ Given that nearly 80% of the participants were free of clinical or subclinical CVDs,^24^ it was plausible that most of them were from stage A. Considering the use of automated office blood pressure (AOBP) in the trial and an approximate 5–10 mmHg difference between AOBP and traditional clinic BP,^26^ a SBP target of less than 130 mmHg was convincing. A meta‐analysis reported that for every 10 mmHg reduction in SBP, the risk of HF was reduced by 28%, which could apply to the subgroup with a mean baseline SBP between 130–140 mmHg.^11^ The results also provided strong evidence for a SBP target of less than 130 mmHg. Furthermore, current HF guidelines recommended BP control for patients at stage A with hypertension according to hypertensive guidelines.^9^, ^27^ Thus, a BP target of less than 130/80 mmHg should be considered.^1^, ^28^, ^29^, ^30^ When it comes to the lower threshold of BP, a post hoc analysis of the China Stroke Primary Prevention Trial (CSPPT) presented that a SBP of less than 120 mmHg was associated with an increasing risk of stroke and all‐cause mortality.^31^ However, it remains unclear due to the lack of robust evidence. We suggest that antihypertensive treatment should be individualized based on tolerability and frailty.

For patients at stage B, a subgroup analysis of the SPRINT study showed that among participants with baseline LVH, intensive BP treatment could significantly regress their LVH,^32^ further preventing the symptomatic HF. A post hoc analysis also specified that a SBP target of less than 130 mmHg was associated with reverse remodeling and improved cardiac function in patients with subclinical hypertensive heart diseases.^33^ In addition, a meta‐analysis found that an intensive BP control of less than 130 mmHg could decrease the risk of HF by 27% in patients with coronary artery disease but without HF. In particular, the 2017 ACC/AHA/HFSA focused update of the 2013 ACC/AHA HF guideline recommended a target of less than 130/80 mmHg for hypertensive patients at high risk of HF as well as hypertensive patients with clinical HF,^34^ which probably could be extrapolated to patients at stage B with hypertension. The target was also consistent with relevant recommendations in the most recent Chinese and Japanese hypertensive guidelines.^1^, ^30^ Overall, we suggest a BP target of less than 130/80 mmHg for patients at stage B with hypertension. However, it is worth noting that achieved BP of less than 120/70 mmHg might be associated with adverse cardiovascular outcomes and caution should be taken if BP falls below this level.^35^, ^36^

For patients at stage C, there are no clinical trials that are designed to compare different BP lowering targets in HF populations. Therefore, the evidence of BP target mainly stems from trials that compared BP lowering drugs to placebo or other active antihypertensive treatment. The Candesartan in HF: Assessment of Reduction in Mortality and morbidity study (CHARM)‐preserved program presented that compared with placebo, a lower BP of less than 130/80 mmHg in the candesartan subgroup significantly prevented admissions for chronic HF.^37^ Aldosterone Receptor Blockade in Diastolic Heart Failure (Aldo‐DHF) study also showed a signal that lowering BP to less than 130/80 mmHg might improve left ventricular diastolic function.^38^ Additionally, the CHARM‐Alternative trial proposed candesartan could reduce the risk of combined cardiovascular death or HF hospitalization by 23% in HFrEF patients with a BP lower than 130/80 mmHg.^19^ A meta‐analysis indicated that a 10 mmHg reduction in SBP could decrease the rate of major CVDs by 34% in patients with a history of HF.^11^ A similar conclusion was seen in another systematic review.^39^ In a position paper by the European Society of Hypertension, it was recommended that BP should be lowered to less than 140/90 mmHg, if tolerated, less than 130/80 mmHg in hypertensive patients with HFpEF.^40^ Notably, the 2017 ACC/AHA/HFSA focused update of the 2013 ACC/AHA HF guideline also recommended a target of less than 130/80 mmHg for patients at stage C with HFpEF and HFrEF.^34^ In line with current evidence, a BP target of less than 130/80 mmHg is reasonable for patients at stage C with hypertension. As for the lower threshold of BP, it remains unclear due to lack of evidence. Considering the definite benefit of guideline‐directed medical therapy (GDMT) and the unclear relationship between lower BP and adverse cardiovascular outcomes,^41^, ^42^ we suggest that lower BP should not deter the up‐titration of GDMT that is shown to improve clinical outcomes unless the intolerance of patients or the occurrence of adverse events.^43^ However, there is little sufficiently robust evidence to ascertain the conclusion and clinical trials focusing on the BP target in these populations are warranted.

For patients at stage D, who always suffer from a loss of myocardial contractility and pump failure, and presumably manifest as reduced cardiac output and systemic congestion.^44^ Thus, hypertension rarely occurs at this stage, even in patients with a history of hypertension.^45^ In contrast, frequent SBP ≤ 90 mmHg serves as one of the clinical indicators of advanced HF.^9^ Several clinical trials reported that the mean BP levels among patients at stage D were relatively lower than those at other stages.^46^, ^47^, ^48^ Sustained hypotension could cause hypoperfusion of the end organs, such as the brain and kidney, leading to end‐organ dysfunction.^44^ In the Carvedilol Prospective Randomized Cumulative Survival (COPERNICUS) trial, patients with advanced HF were enrolled and classified by pretreatment SBP. The results showed that patients with lower pretreatment SBP were at higher risk of a major clinical event, regardless of treatment.^49^ In fact, relatively higher BP for patients at stage D may represent an opportunity to maximize GDMT and indicate a better prognosis.

### 1.4 Management of hypertension according to stages of HF

Given the poor prognosis of clinical HF, primary prevention is the most important step that should be realized at stage A and stage B. Measures to prevent the progression of clinical HF, especially BP control, are of important priority at these stages. Considering the high prevalence of hypertension and the potential benefit from antihypertensive treatment in Asian populations, it is imperative to take measures to improve BP control. From the perspective of antihypertensive treatment, it should be emphasized that BP reduction is more important than the choice of specific BP‐lowering regimens.^50^ Nonetheless, there is evidence of mildly distinct effects in preventing HF among different BP‐lowering drugs. In a large meta‐analysis, calcium channel blockers (CCBs) seemed to be inferior to other regimens for the prevention of HF, whereas diuretics were superior to other drug classes.^11^ Another meta‐analysis ascertained the finding that CCBs were significantly less effective in preventing HF than the other four classes of drugs with a relative risk of 1.22 (95% confidence interval, 1.10 to 1.35).^51^ The Antihypertensive and Lipid‐Lowering Treatment to Prevent Heart Attack Trial (ALLHAT) also found that thiazide‐type diuretics were superior to angiotensin‐converting enzyme inhibitors (ACEIs) and CCBs in preventing HF. According to the 2022 AHA/ACC/HFSA HF guideline, ACEIs, angiotensin receptor blockers (ARBs) and β‐blockers were recommended for preventing HF in patients with structural or functional heart diseases,^52^, ^53^, ^54^ which are in accordance with hypertensive guidelines.^30^

In addition to conventional antihypertensive drugs, angiotensin receptor‐neprilysin inhibitor (ARNI) is a new class of cardiovascular agent that shows a tremendous antihypertensive effect, even towering over ARBs.^55^, ^56^ The apparent clinical benefit of ARNI in patients with HF was demonstrated by clinical trials.^41^ However, whether this agent could prevent HF in hypertensive patients is uncertain and further clinical trials are needed to investigate it. Sodium‐glucose cotransporter‐2 (SGLT2) inhibitors, developed initially as a hypoglycemic agent, have shown great benefit in reducing the risk of HF in patients with type 2 diabetes.^57^ The potential mechanisms involved include promoting natriuresis and osmotic diuresis, reducing sympathetic hyperactivity and chronic inflammation, and improving glucose metabolism and obesity.^58^ It is generally acknowledged that Asian populations have higher salt intake and salt sensitivity than western populations.^2^ SGLT2 inhibitors could significantly reduce nocturnal BP, especially for patients with high salt sensitivity, ultimately helping reduce the risk of HF.^59^ Besides, over the past two decades, urbanization as well as changes in diet and lifestyle in many Asian countries and regions have led to the epidemic of obesity.^60^ More and more hypertensive patients suffer from obesity. Both hypertension and obesity could cause LVH and HF through overlapping neurohormonal pathways, such as activation of the renin‐angiotensin‐aldosterone system.^61^ Due to the multiple pharmacologic actions of SGLT2 inhibitors, they could improve the control of BP and weight along with their hypoglycemic effect. Based on the evidence, we recommend the use of SGLT2 inhibitors for patients with diabetes complicated with hypertension at stage A and stage B. Furthermore, glucagon‐like peptide‐1 receptor (GLP‐1R) agonists also lead to significant weight loss and BP reduction, which might serve as potential drugs for overweight or obese patients with hypertension.^62^

For patients at stage C, most drugs that are recommended to treat HF can lower BP simultaneously. ACEIs, ARBs, β‐blockers and mineralocorticoid receptor antagonists (MRAs) were recommended as first‐line therapy for patients with HFrEF to reduce mortality and HF hospitalization, whereas diuretics were suggested to relieve the symptoms of congestion.^9^, ^22^, ^63^ In particular, ARNI and SGLT2 inhibitors were listed as first‐line therapy recently based on strong evidence from large randomized clinical trials (RCTs). Compared with enalapril, ARNI reduced the risk of mortality and HF hospitalization in HFrEF patients by 16% and 21%, respectively.^41^ Dapagliflozin and empagliflozin were also demonstrated to be able to lower the risk of cardiovascular death and HF hospitalization in HFrEF patients, irrespective of diabetes.^42^, ^64^ As for CCBs, they are not recommended for the treatment of HF due to the lack of direct benefit. However, amlodipine and felodipine appear to be safe in patients with HF, which can be added to patients who do not reach the BP goal despite GDMT.^65^, ^66^ Additionally, a combination of hydralazine and isosorbide dinitrate can be considered in blacks or in patients with HFrEF who cannot tolerate renin‐angiotensin system antagonists, but no conclusive evidence has been found in Asian populations and further studies are needed.^67^, ^68^

For patients with HFpEF, diuretics are recommended to improve symptoms caused by congestion.^22^, ^63^ Several large RCTs were designed to examine the treatment effects of different drugs, including ACEIs, ARBs, ARNI, β‐blockers, MRAs, and SGLT2 inhibitors.^20^, ^37^, ^69^, ^70^, ^71^, ^72^, ^73^ Notably, only empagliflozin (Empagliflozin in Heart Failure with a Preserved Ejection Fraction trial) could significantly reduce the incidence of its primary endpoint of the combined risk of cardiovascular death or hospitalization for HF by 21%.^69^ However, the effects were mainly associated with the decrease in the rate of hospitalization for HF, rather than cardiovascular death. Besides, hospitalization for HF was also significantly reduced by candesartan and spironolactone,^37^, ^72^ and there was a borderline benefit for a reduction of hospitalization for HF with sacubitril‐valsartan.^73^ Although a few large‐scale observational studies presented the benefit of renin‐angiotensin system antagonists and β‐blockers in lowering all‐cause mortality,^74^, ^75^ RCTs failed to demonstrate it.

For patients at stage D, relatively high BP indicates an opportunity to maximize GDMT. We suggest titrating the doses of ACEIs/ARBs/ARNI, β‐blockers, MRAs, and SGLT2 inhibitors with caution, if tolerable. In addition, measures should be taken to improve hemodynamics. Fluid restriction, inotropic support, mechanical circulatory support, and cardiac transplantation might be considered.^9^ The summary of the management of HF according to stages is displayed in Table 2.

**TABLE 2:** Summary of the management of hypertension according to stages of heart failure

Stages of HF	Stage A (At risk for HF)	Stage B (Pre‐HF)	Stage C (Symptomatic HF)	Stage D (Advanced HF)
BP targets	<130/80 mmHg	<130/80 mmHg	<130/80 mmHg	
Drugs beneficial	ACEI, ARB, β‐blockers, diuretics	ACEI, ARB, β‐blockers, diuretics	**HFrEF**: ACEIs, ARBs, ARNI, β‐blockers, MRAs and SGLT2 inhibitors **HFpEF**: SGLT2 inhibitors, ARBs, MRAs, ARNI	**HFrEF**: ACEIs, ARBs, ARNI, β‐blockers, MRAs and SGLT2 inhibitors **HFpEF**: SGLT2 inhibitors, ARBs, MRAs, ARNI
Drugs with limited benefit	CCB	CCB	**HFrEF**: CCB **HFpEF**: ACEIs, β‐blockers, CCB	**HFrEF**: CCB **HFpEF**: ACEIs, β‐blockers, CCB
Potential drugs	SGLT2 inhibitors, ARNI	SGLT2 inhibitors, ARNI		
Others	Special caution is needed if SBP <120 mmHg.	Special caution is needed if BP <120/70 mmHg.	Lower BP should not deter the up‐titration of GDMT, if tolerable and without adverse events. Diuretics are recommended to improve symptoms.	Measures should be taken to improve hemodynamics.

Abbreviations: ACEI, angiotensin converting enzyme inhibitor; ARB, angiotensin receptor blocker; ARNI, angiotensin receptor neprilysin inhibitor; BP, blood pressure; CCB, calcium channel blocker; GDMT, guideline‐directed medical therapy; HF, heart failure; HFpEF, heart failure with preserved ejection fraction; HFrEF, heart failure with reduced ejection fraction; MRA, mineralocorticoid receptor antagonist; SGLT2, sodium‐glucose cotransporter‐2.

## 2 CONCLUSION

Hypertension is an important risk factor as well as a common comorbidity of HF. Given the high prevalence of hypertension as well as HF in Asia, the management of hypertension is a priority for preventing and alleviating the development of HF. In the early diagnosis of HF with hypertension, the development of AI methods including the integration of more parameters that can be easily obtained by simple diagnostic methods can be helpful. Focusing on stages of HF, we propose the BP target of less than 130/80 mmHg for patients at stage A, B, and C with hypertension. For patients at stage D, relatively higher BP may represent an opportunity to maximize GDMT and indicate a better prognosis. Traditional antihypertensive drugs are still the cornerstones for the management of hypertension at stage A and stage B. For patients at stage C with hypertension, GDMT is essential as it also helps the control of BP. Notably, SGLT2 inhibitors are newer regimens recommended for the treatment of HF and presumably even in hypertension to prevent HF. In terms of patients at stage D, GDMT is also recommended if tolerable.

## CONFLICT OF INTEREST

The authors have no competing interests.

## AUTHOR CONTRIBUTIONS

Huanhuan Miao: Designed the study, carried out the literature search, performed the analysis and wrote the manuscript. Changhong Zou: Designed the study, performed the analysis and revised the manuscript. Shijie Yang: Carried out the literature search, designed the tables and revised the manuscript. Yook‐Chin Chia, Minh Van Huynh, Guru Prasad Sogunuru, and Jam Chin Tay: Revised the manuscript. Tzung‐Dau Wang: Designed the tables and revised the manuscript. Kazuomi Kario: Designed the studyand revised the manuscript. Yuqing Zhang: Designed the study, performed the analysis and revised the manuscript.
